# Metabolomics analysis of stool in rats with type 2 diabetes mellitus after single-anastomosis duodenal–ileal bypass with sleeve gastrectomy

**DOI:** 10.3389/fendo.2022.1013959

**Published:** 2022-09-20

**Authors:** Lun Wang, Zeyu Wang, Yang Yu, Zhaoheng Ren, Yongheng Jia, Jinfa Wang, Shixing Li, Tao Jiang

**Affiliations:** Department of Bariatric and Metabolic Surgery, China-Japan Union Hospital of Jilin University, Changchun City, China

**Keywords:** single-anastomosis duodenal–ileal bypass with sleeve gastrectomy, type 2 diabetes mellitus, metabolomics, bariatric surgery, SADI-S, T2DM

## Abstract

**Background:**

Single-anastomosis duodenal-ileal bypass with sleeve gastrectomy (SADI-S) is one of the most effective bariatric procedures in the treatment of type 2 diabetes mellitus (T2DM). However, the mechanisms by which SADI-S improves T2DM are not well-known.

**Objective:**

To explore the effects of SADI-S on metabolites in the stool of rats with T2DM.

**Methods:**

Twenty rats were fed on high-fat diet and administered with a low-dose (30mg/kg) of streptozotocin to establish T2DM models. The rats were then randomly assigned to the SADI-S group (n=10) and sham operation group (n=9). Stool samples were collected from all rats at 8 weeks after surgery and stored at -80 °C. Metabolomics analysis was performed to identify differential metabolites through ultra- performance liquid chromatography-mass spectrometry.

**Results:**

At 8-week after surgery, rats of the SADI-S group showed significantly decreased fasting blood glucose, glucose tolerance test 2-hour, glycated haemoglobin, and body weight compared with those of the sham group. A total of 245 differential metabolites were identified between the two groups, among which 8 metabolites were detectable under both the positive ion model and negative ion model. Therefore, a total of 237 differential metabolites were identified in our study which were mainly involved in tryptophan metabolism; cysteine and methionine metabolism; phenylalanine metabolism; phenylalanine; tyrosine and tryptophan biosynthesis; arginine biosynthesis; alanine, aspartate and glutamate metabolism; Arginine and proline metabolism; glyoxylate and dicarboxylate metabolism; alpha-Linolenic acid metabolism; Linoleic acid metabolism; riboflavin metabolism; nicotinate and nicotinamide metabolism; pyrimidine metabolism; porphyrin and chlorophyll metabolism.

**Conclusion:**

SADI-S significantly improved the glucose metabolism in T2DM rats. In addition, SADI-S significantly changed the composition of metabolites in T2DM rats which were involved in tryptophan metabolism pathway, linoleic acid metabolism pathway and so on. This may be the mechanism by which SADI-S improved T2DM.

## Introduction

With the rapid development of social economy and changes in people’s lifestyles, the number of people with diabetes has been increasing yearly. According to the latest report by the International Diabetes Federation, the global number of patients with diabetes (aged 20-79 years) was 537 million, and there were 6.7 million deaths due to diabetes in 2021. Moreover, the total health expenditure on diabetes managements was more than 966 billion US dollars in 2021 (1). Approximately, 90% of all patients with diabetes are of type 2 diabetes mellitus (T2DM). Traditional therapeutic strategies, including diet control, exercise, and the utilization of hypoglycemic agents, have not been successful in improving T2DM. Compared with traditional therapeutic strategies, bariatric surgery has been shown to effectively treat T2DM (2–4). For this reason, bariatric surgery is routinely applied in the treatment of T2DM patients.

As one of the currently used bariatric procedures, single-anastomosis duodenal–ileal bypass with sleeve gastrectomy (SADI-S) was first established in 2007 by Torres et al. based on the principle of biliopancreatic diversion with duodenal switch (BPD/DS) (5). SADI-S and BPD/DS are widely acknowledged as the most effective bariatric procedure for T2DM treatment (6–8). Compared with BPD/DS, SADI-S is preferred because it not only maintains similar effects in weight loss and remission of metabolic diseases of BPD/DS, but has a lower operative and malnutritional risk given its ability to reduce one anastomosis and lengthen the common channel of intestine.

Metabolomics is an effective method for quantifying the metabolites of an organism and determine association of metabolites with physiological and pathological changes (9, 10). It has been applied to explore the mechanisms underlying efficacy of bariatric surgery (11). However, the impacts of SADI-S on metabolites in T2DM are currently unknown.Therefore, this study was conducted to explore the effects of SADI-S on metabolites in stool of rats with T2DM, and determine whether changes in metabolites affect the surgery-induced T2DM remission.

## Materials and methods

### T2DM animal model and groups

Twenty male Wistar rats (8 weeks old) were purchased from the Vital River Laboratory Animal Technology Co., Ltd (Beijing, China). The rats were housed in individual cages under constant ambient temperature and humidity controlled to a 12-h day/night cycle. After 2-week adaptive feeding with an ordinary feed (containing 13.8% fat, 63.4% carbohydrate, and 22.8% protein), the rats were fed with a high-fat diet (containing 45.6% fat, 37.9% carbohydrate, and 16.5% protein) for 8 weeks to induce insulin resistance. 100 mg of streptozotocin (STZ) (Sigma, USA) were dissolved in 10 ml of ice-cold citrate buffer (0.1 mol/L, PH=4.5) to prepare a STZ solution with a concentration of 10 mg/mL. The rats were fasted for 12 h and intraperitoneally injected with STZ (30 mg/kg).

The T2DM rat model was considered successful if rats’ random blood glucose was at least 16.7 mmol/L after 72 h from STZ injection. Based on this criterion, the T2DM rat model was successfully established in 19 rats randomly assigned to the SADI-S group (n =10) and sham operation group (n =9). Three weeks after STZ injection, SADI-S and sham operation were performed. All animal protocols were approved by the Animal Experiment Ethics Committee of First hospital of Jilin University, and all rats were cared according to the national guidelines for the care of animal of the People’s Republic of China.

### Preoperative preparation

The night before surgery, rats were placed in a cage with a raised wire platforms to avoid coprophagy and fasted for approximately 12 h. To prevent hyperglycemia which will increase the risk of surgery, blood glucose was regularly measured before surgery. An appropriate dose of insulin was injected subcutaneously if blood sugar was too high. The rats were anaesthetized through intraperitoneal injection of pentobarbital sodium solution (10 mg/mL).

The abdominal fur of rat was shaved from the sternum to groin using an electric hair clipper. Part of the dorsal and femoral fur were also shaved for hydration and injection of antibacterial agents, respectively. Hydration was performed by subcutaneously injecting 10 mL 0.9% saline solution from multiple sites before beginning surgery.

### Surgical procedures

The SADI-S surgery was performed according to the protocol described by Wang et al. [ (12)]. Briefly, two thirds of the stomach along with the greater curvature were removed. Secondly, we transected the duodenum about 5 mm from the pylorus, and sutured the duodenal stump using a 6-0 PDS suture (Ethicon). Thirdly, we retrogradely measured a 40-cm small intestine from the ileocecal junction and marked it with sutures. Fourthly, we performed an end-to-side anastomosis of the proximal duodenum with the marked ileal at 40-cm away from ileocecal junction. Finally, the abdomen was closed using a 4-0 non-absorbable silk suture. Rats in the sham operation group received laparotomy only.

### Postoperative care

At the end of surgery, the rats were immediately placed on a heating pad in a prone position until they fully regained consciousness. They were then subcutaneously injected with 30 mL parenteral nutrition solution (20ml 50%GLU+ 10ml calcium gluconate+70ml 0.9%NaCl solution+3U insulin) daily in the first 3 postoperative days (10 mL at 7:00am,10 mL at 3pm and 10 mL at 11pm). Meanwhile, ceftazidime (90 mg/kg) was administered intramuscularly to prevent infection in the first 3 postoperative days. In terms of feeding, we gradually decreased the amount of liquid diet and increased the amount of solid diet at day 4-6 postoperatively. Full solid diet was provided on day 7 postoperatively. The postoperative food intake per day was similar in both groups.

### Intraperitoneal glucose tolerance test

The IPGTT was performed before surgery and at 8 weeks after surgery. After fasting for 12 h, rats were intraperitoneally injected with 50% glucose (2g/kg). Subsequently,we measured blood glucose *via* pricking the tail vein before injection and at 15,30, 60, 120, and 180 min after injecting glucose. Finally, a blood glucose-time curve was constructed and used to calculate the area under the curve (AUC) of IPGTT.

### Histological assessment

Eight weeks after surgery, the pancreas and small intestine tissue samples of rats were fixed in paraformaldehyde solution,embedded, cut into 4 μm sections, stained with immunofluorescence double-labeling staining or hematoxylin and eosin (H&E),followed by observing the pathological changes in the pancreas and small intestine using a light microscope.

### Non-targeted metabolomics analysis

#### Sample preparation

At 8 weeks after surgery, stool samples were collected by stimulating the rats’ anus with a sterile pincer. The samples were immediately placed in liquid nitrogen and stored at -80°C until use. To identify metabolites by liquid chromatography-mass spectrometry, stool samples were processed as follows: a) Stool samples were thawed on ice; b) 50 mg of stool samples and 800 µl of 80% methanol were mixed in a labeled 1.5-ml microcentrifuge tube; c) the specimens were ground at 65 HZ for 90 s, thoroughly mixed on a vortex mixer, sonicated for 30 min at 4°C, allowed to stand for 1 h at -40°C, vortexed for 30 s, allowed to stand for 30 min at 4°C, and centrifuged at 12,000 rpm at 4°C for 15 min. Next, 200 uL of the supernatant and 5 uL of the internal standard (0.14 mg/mL dichlorophenylalanine) were added into a labeled 1.5-ml microcentrifuge tube, mixed and transferred into autosampler vial.

#### Liquid chromatography-mass spectrometry conditions

The stool samples were subjected to LC-MS (Waters, UPLC; Thermo, Q Exactive) to separate metabolites. The chromatographic separation conditions were set as follows: Column temperature: 40°C; Flow rate: 0.3 mL/min; Mobile phase A: water +0.05% formic acid; Mobile phase B: acetonitrile; Injection volume: 5 ul; Automatic injector temperature: 4°C. The following elution program was used: 0.00-1.00 min, 0.3 mL/min, 95% A,5% B;1.00-13.50 min, 0.3 mL/min, 5% A, 95% B; 13.50-16.00 min, 0.3 mL/min, 95% A,5% B. The conditions of electrospray ionization (ESI) source were set as follows: ESI+: Heater Temp 300°C; Sheath Gas Flow rate, 45 arb; Aux Gas Flow Rate, 15 arb; Sweep Gas Flow Rate, 1arb; spray voltage, 3.0KV; Capillary Temp, 350°C; S-Lens RF Level, 30%. ESI-: Heater Temp 300°C, Sheath Gas Flow rate,45arb; Aux Gas Flow Rate, 15arb; Sweep Gas Flow Rate, 1arb; spray voltage, 3.2KV; Capillary Temp, 350°C; S-Lens RF Level, 60%.

#### Multivariate data processing and analysis

The R package XCMS (version 3.3.2) was used to process the raw data in terms of peak identification, filtration, and alignment. The MS2 database was used to identify the differential metabolites. The quality of data was determined based on the relative standard deviation (RSD). The data was considered to have a high quality when the proportion of characteristic peaks with RSD<30% was at least 70%. The data were analyzed using multivariate statistical analyses, including Principal Component Analysis (PCA) and Orthogonal Projections to Latent Structures Discriminant Analysis (OPLS-DA). Clusters and differences between SADI-S group and sham operation group were determined by PCA and OPLS-DA. The R^2^Y and Q^2^ values were used to evaluate the stability and predictability of each model. Differential metabolites were defined as those metabolites with P ≤ 0.05 and variable importance in the projection (VIP)≥1. A Permutations Plot was constructed to evaluate over-fitting in the PLS-DA model.

#### Statistical analysis

Statistical analysis was performed using SPSS 22.0 software. All data are presented as mean ± standard deviation, and differences between groups were compared using wilcoxon rank sum test. Differences were considered statistically significant at a P value less than 0.05.

## Results

### Surgical outcomes

Five T2DM rats died during the study. In the SADI-S group, four rats died after operation, 3 due to bleeding and 1 rat because of anastomotic leakage. Finally, 6 rats survived to 8 weeks after SADI-S surgery until they were sacrificed. In the sham operation group, one rat died due to incision rupture at day 12 postoperatively and 8 rats survived to 8 weeks after surgery until they were sacrificed.

### Changes in body weight and glucose metabolism

**Figure 1:**
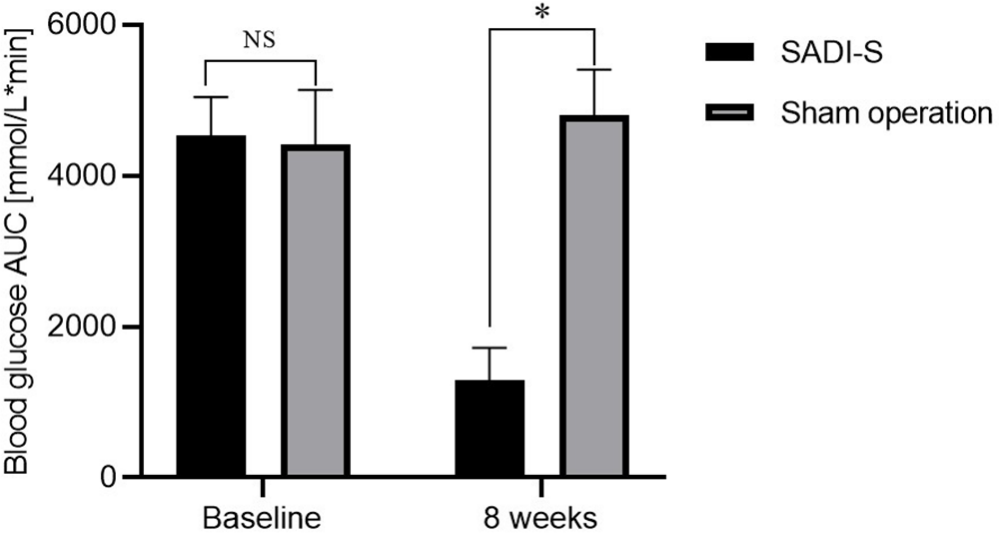
Changes in body weight **(A)**, changes in blood glucose AUC during IPGTT before and after surgery **(B)** and FBG **(C)** before and after surgery. Comparison of the HBA1c level at 8 weeks after surgery between the two groups **(D)**. All values are expressed as Mean ± SD. NS indicates no significant difference between the SADI-S group and sham operation group. * represents a significant difference between the SADI-S group and sham operation group (P< 0.05).

The changes in body weight are illustrated in **Figure 1A**. Notably, the SADI-S surgery resulted in a significant decrease in body weight (*P*<0.05). The body weight in the sham operation group also showed decreasing trend. However it was observed that the decrease in body weight at each postoperative follow-up point was significantly higher in the SADI-S group compared with the sham operation group (*P*<0.05).

The changes in area under curve (AUC) of blood glucose during IPGTT and fasting blood glucose (FBG) before and after surgery are illustrated in **Figures 1B, C**. The levels of FBG and AUC values for glucose were significantly decreased after SADI-S surgery compared with the preoperative levels. In contrast, the FBG and AUC values for glucose were increased in the sham operation group. At 8 weeks after surgery, the level of glycated hemoglobin (HBA1c) in the SADI-S group was significantly lower compared with level in the sham operation group (**Figure 1D**).

### Histological assessment of pancreas and small intestine

**Figure 2:**
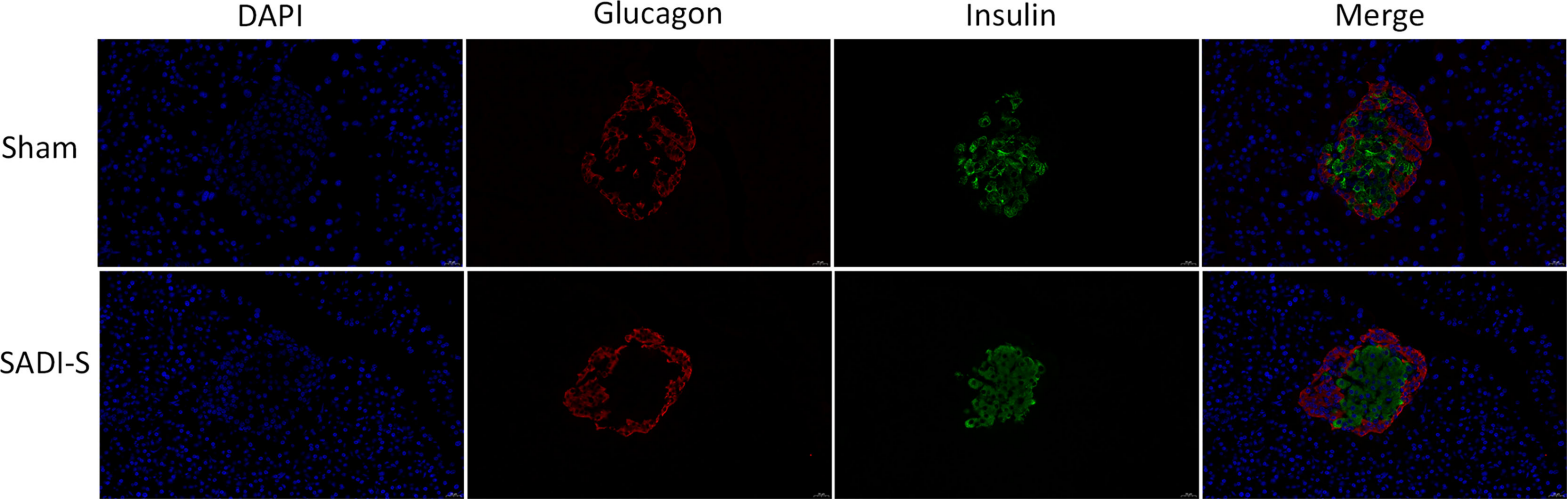
Immunofluorescence double-labeling staining images. Morphological and histological evaluation of pancreas tissues between the SADI-S group and the sham operation group. Red represents pancreatic islet α cells, green represents pancreatic islet β cells, and blue represents cell nucleus.

The immunofluorescence double-labeling staining images of the pancreas tissue are shown in **Figure 2**. Significant pancreatic islet β cells damage accompanied by marked degeneration of vacuolation were observed in the sham group compared with the SADI-S group. In addition, the distribution of pancreatic islet β cells and α cells disorderly in the sham group. In contrast, the distribution pattern of β cells and morphology in the SADI-S group was uniform and there was no significant vacuolar degeneration.

**Figure 3:**
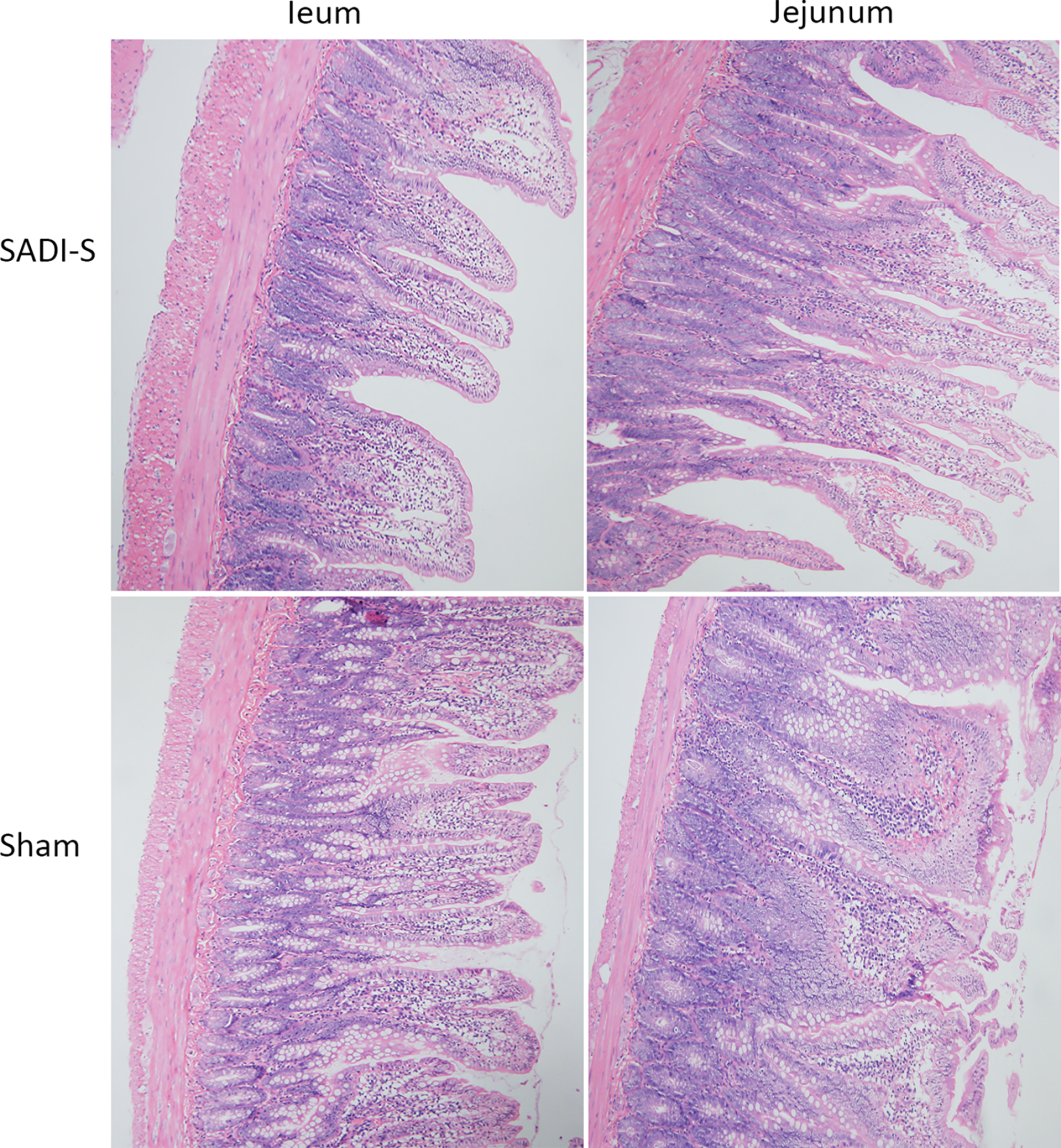
Hematoxylin and eosin (H&E) staining photographs. Morphological and histological evaluation of ileum and jejunum between the SADI-S group and the sham operation group (200×).

The hematoxylin and eosin (H&E) staining images of the small intestine are shown in **Figure 3**. In the SADI-S group, the structure of the small intestinal mucosa was normal, with tall and continuous villi arranged neatly, whereas the villi of small intestine in the sham operation group,especially in the ileal, was swollen, short, thick and disorderly, some villi were necrotic, shed or disappeared.

### Metabolomics analysis

#### Multivariate statistical analysis

**Figure 4:**
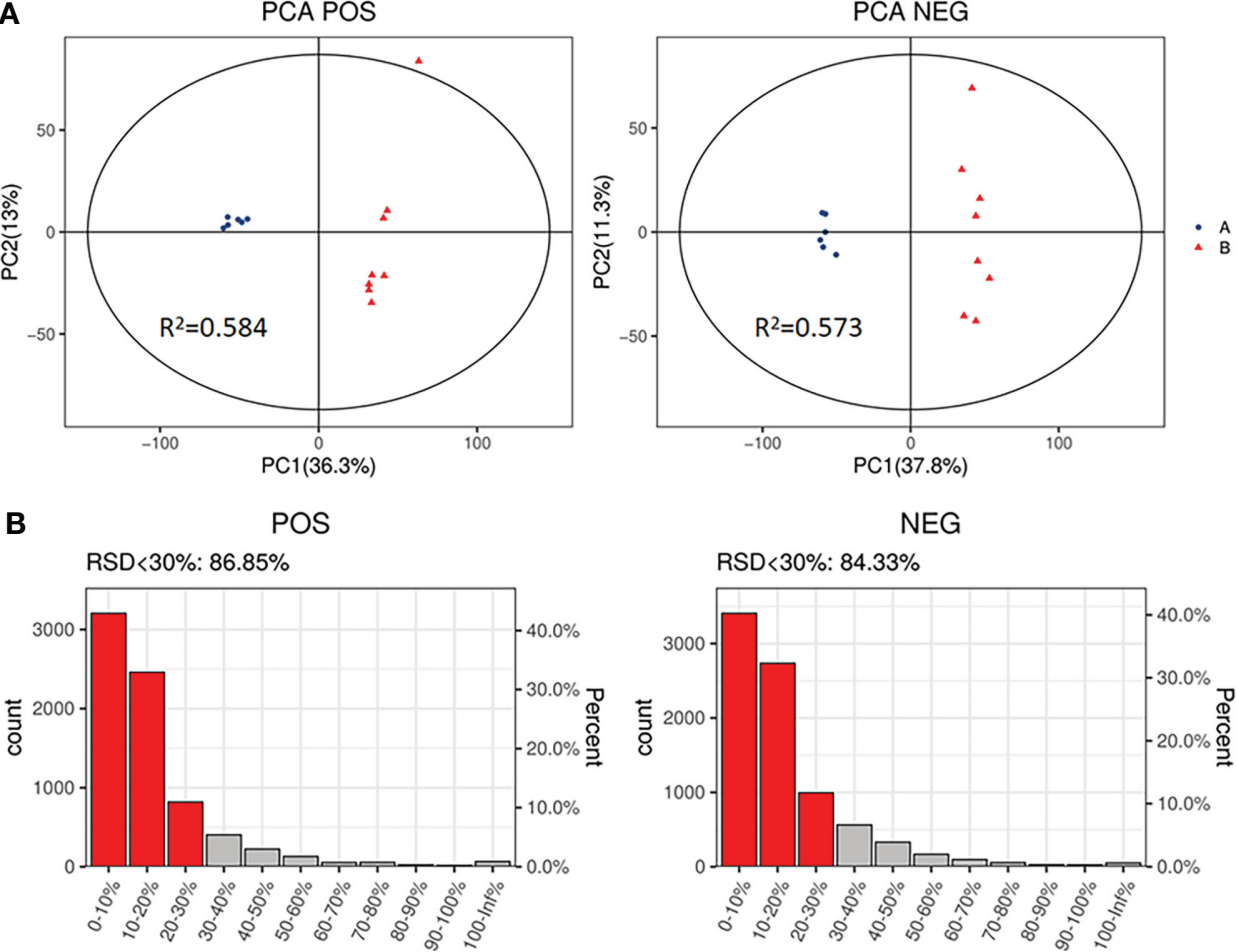
PCA score plot of SADI-S group and sham operation group in the positive ion mode and in the negative ion mode **(A)**; quality assurance in the positive ion mode and in the negative ion mode **(B)**. A=SADI-S group, B=sham operation group.

The PCA, an unsupervised analysis method, was used to provide a comprehensive view of stool samples. The parameter R^2^X and percent of relative standard deviation (RSD)<30% are often used to reflect the stability of the LC-MS system and the reliability of data. As shown in **Figures 4A, B**, the R^2^X>0.5 was obtained both in the positive ion mode and the negative ion mode, and percent of RSD<30% exceeded 70% in both modes. These results indicated that the LC-MS system was highly stable for metabolomics analysis and the data were reliable. Besides, analysis of the results revealed that the metabolic profile of the SADI-S group was different from that of the sham-operated group, indicating that the metabolites in stool of T2DM rats were significantly changed after SADI-S.

**Figure 5:**
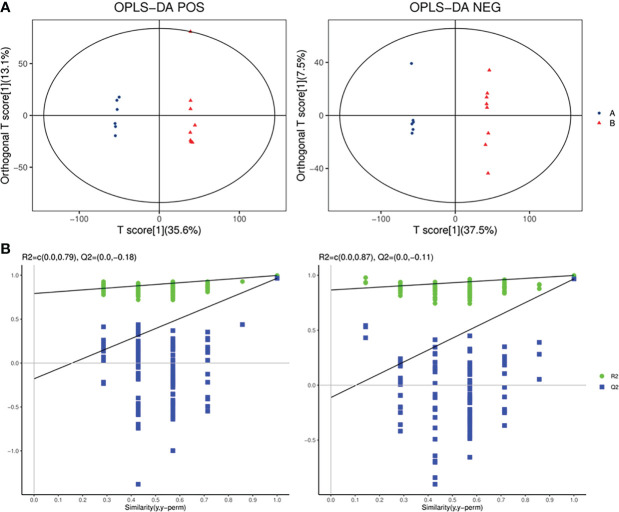
The plot of OPLS-DA score for SADI-S group and sham operation group in the positive ion mode and in the negative ion mode **(A)**; permutations plot in the positive ion mode and in the negative ion mode **(B)**. A=SADI-S group, B=sham operation group.

OPLS-DA, a supervised analysis method, was performed to analyze differences between the SADI-S group and sham operation group. As shown in **Figure 5A**, the OPLS-DA score plot revealed that the metabolic profile of T2DM rats treated with SADI-S was significantly different from that of sham operation group both in the positive ion mode and in the negative ion mode, indicating that SADI-S significantly altered the metabolic profile of T2DM rats. The two classification parameters, R^2^Y and Q^2^, were 0.998 and 0.966 in the positive mode,  0.999 and 0.968 in the negative mode, respectively. The R^2^Y and Q^2^ were >0.5, suggesting that the OPLS-DA model had good stability and predictability. Analysis of the Permutations Plot shown in **Figure 5B** indicated that the intersection between the regression line of Q^2^ point and the ordinate was negative value, implying that there was no over-fitting in the OPLS-DA model.

#### Identification of differential metabolites

According to the OPLS-DA model, VIP was generated to illustrate the importance of variables in explaining the X dataset and the associated Y dataset. The screening conditions of differential metabolites were as follows: VIP≥1 and p-value ≤ 0.05. The data shown in the **Supplemental Table 1** indicated that 245 differential metabolites were identified, among which 81 metabolites were obtained under the positive ion model and 164 metabolites were obtained under the negative ion model. Eight of these metabolites were detectable in both modes. Therefore, a total of 237 differential metabolites were identified between the two groups. Notably, 181 metabolites were significantly higher in the SADI-S group than in the sham operation group. Moreover, 64 metabolites were significantly lower in the SADI-S group than in the sham operation group. According to the previous literature, some metabolites that may be associated with the T2DM such as branched-chain amino acids (valine), aromatic amino acid (phenylalanine), bile acid (cholic acid, lithocholic acid and b-muricholic acid), short-chain fatty acid (isobutyric acid), phospholipid lysoPE(17:0), lysoPE (20:3), and lysoPS(16:0), were also observed to have a significant changes in our study.

To explore the direct relationship between the groups, we constructed a heatmap based on the abundance value of each metabolite. The **Supplemental Figure 1** and **Figure 2** show the overall changes in the common features and the tendency of variation in metabolite levels in both groups. In the heatmap, the positions of metabolites with similar changes in abundance were closer and the abundance of metabolites in T2DM rats treated with SADI-S was significantly difference from that of the sham group.

#### Pathway analysis of differential metabolites

**Figure 6:**
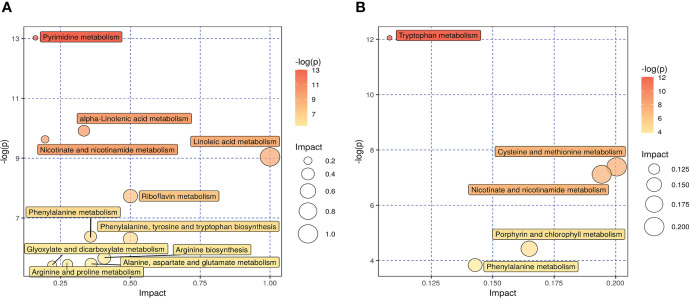
The effect of SADI-S on metabolic pathway in the negative ion mode **(A)** and in the positive ion mode **(B)**. The bigger the circle, the more significant is the enrichment. The darker the yellow, the smaller the P value.

Kyoto Encyclopedia of Genes and Genomes (KEGG) analysis was performed to screen differential metabolic pathways based on the following conditions: pathway impact >0.1 and P < 0.05 (13). As shown in **Figures 6A, B**, the following 14 metabolic pathways were identified in SADI-S group: tryptophan metabolism; cysteine and methionine metabolism; phenylalanine metabolism; phenylalanine; tyrosine and tryptophan biosynthesis; arginine biosynthesis; alanine, aspartate and glutamate metabolism; Arginine and proline metabolism; glyoxylate and dicarboxylate metabolism; alpha-Linolenic acid metabolism; Linoleic acid metabolism; riboflavin metabolism; nicotinate and nicotinamide metabolism; pyrimidine metabolism; porphyrin and chlorophyll metabolism.

## Discussion

To the best of our knowledge, the current study was the first to explore the effects of SADI-S on metabolites of stool in T2DM rats. The results of the present study showed that SADI-S significantly lowered the blood glucose level and altered the level of 245 metabolites in T2DM rats. This was evaluated using a low-dose STZ and HFD. Among the 245 metabolites, 16 of them were associated with the remission of T2DM after SADI-S.

Branched-chain amino acids (BCAAs) are correlated with the occurrence of T2DM and are significantly elevated in T2DM (14–17). Three potential mechanisms for how BCAAs lead to the occurrence of T2DM are: First, increased levels of BCAAs can result in the accumulation of toxic metabolites, which in turn causes mitochondrial dysfunction and a decrease in secretion of insulin in pancreatic beta cells (18, 19); second, perturbations of BCAAs can induce insulin resistance through the mTOR signaling pathway (20); third, increased levels of BCAAs can activate the rapamycin pathway, which then disturb the action of insulin and promote the degradation of insulin receptor substrates (21, 22). A previous study found that the level of valine was significantly higher in patients with T2DM as compared with healthy individuals (23). Several studies have reported that bariatric surgery can cause a significant decrease in levels of BCAAs in T2DM (24, 25). Consistent with these studies, we found that the level of Valine, a type of BCAAs, was significantly reduced in T2DM after SADI-S.

Phenylalanine is an aromatic amino acid which is involved in sugar metabolism after it is oxidized to tyrosine by phenylalanine hydroxylase (26). Previous studies have shown that the level of phenylalanine is significantly decreased in patients with diabetes (27, 28). In the current study, SADI-S caused a significant elevation in the level of phenylalanine and also remission of T2DM.

Tryptophan and its derivates, especially those from the kynurenine and serotonin pathways, are also correlated with glucose metabolism (29–32). Kynurenic acid is a metabolite of tryptophan, derived from the kynurenine pathway and is closely related with the occurrence of T2DM (33–35). Previous studies have shown that bariatric surgery causes a decrease in level of kynurenic acid accompanied with diabetes remission (36, 37). Xanthurenic acid is a metabolite of tryptophan which has been found to be conductive to development of diabetes through chelating complexes with insulin and hence inducing pathological apoptosis of pancreatic β cells (38). 5-hydroxytryptophan acid is another a kind of tryptophan derivative which forms hydrogen bonds with the key residues of the PPAR-r protein at its indole ring and carboxyl group. A separate study has shown that 5-hydroxytryptophan acid can be used as a lead compound to develop the PPAR-r agonists (39). In the present study, SADI-S showed a significant therapeutic effect in T2DM comparable to the effects noted in the previous studies. In addition, it has been shown that the levels of above-mentioned tryptophan derivatives are significantly increased after the SADI-S surgery. However, there is need for further study to evaluate the reasons for the observed difference.

Some studies have shown that low levels of glutamate and glutamine are associated with T2DM (34, 40). In the current study, it was evident that the levels of glutamate and glutamine were significantly increased after SADI-S, which were in consonance with the findings of the previous studies. It has also been found that the level of proline is decreased in patients with diabetes (18). Results of the current study showed that the level of proline was significantly lower in the sham operation group as compared with the SADI-S group. Elsewhere, it has been evident that the level of alanine is elevated after RYGB (41). Similarly, an elevated alanine levels after SADI-S was also found in the present study.

Hippuric acid is synthesized from benzoic acid and glycine in liver. An elevated level of glycine causes an increase in level of hippuric acid and a decrease in level of the branched-chain amino acid, which contribute to the restoration of the mitochondrial function. A previous study has shown that increased level of hippuric acid is accompanied by a decrease in the levels of branched-chain amino acids after Roux-en-Y gastric bypass (41). The present study also showed that the remission of diabetes after SADI-S was accompanied by an elevation in the level of hippuric acid and a decline in the level of branched-chain amino acid.

The alterations in the level of bile acid influences glucose metabolism by acting as the farnesoid X receptors (FXRs). Cholic acid, lithocholic acid, deoxycholic acid, and taurochenodeoxycholic acid are FXR agonists (42). The increased levels of the bile acids activate FXRs and then downregulate the level of blood glucose. Our results showed that the levels of cholic acid and lithocholic acid were significantly increased after SADI-S, while as the levels of deoxycholic acid and taurochenodeoxycholic acid were significantly declined.β-Muricholic acid is a FXRs antagonist (42) and an increase in its levels inhibits FXR and then increases the levels of blood sugar. Our results showed that the levels of β-Muricholic acid were significantly decreased after SADI-S.

Isobutyric acid is a short-chain fatty acid which primarily originate from the degradation of leucine, isoleucine, valine, and proline in gut bacteria (43). Previous study has shown that an elevated level of Isobutyric acid can improve diabetes (44). In agreement with this, the level of isobutyric acid, which is responsible for the diabetes remission, was significantly increased after SADI-S.

The metabolic disorder of phospholipid contributes to development of diabetes (45). It has been shown that LysoPC, lysoPE, and lysoPS are associated with diabetes-related inflammatory stress (18, 45, 46). Therefore, the levels of lysoPC, lysoPE, and lysoPS significantly decreases after diabetes remission. However, the results obtained in the present study showed that the levels of LysoPE (17:0), LysoPE (20:3), and LysoPS (16:0) decreased, whereas the levels of LysoPE (18:2), LysoPE (18:3), LysoPE (17:1) and LysoPC (14:0) were elevated. This was in agreement with the findings of the study by Marta Ugarte et al. (18).

It has been previously found alterations of urea cycle intermediates and urea cycle enzymes in diabetic rats induced by STZ and HFD (18). Ornithine is an intermediate of urea cycle which is synthesized from L-arginine in liver, and its levels are reduced in patients with diabetes (47). It promotes the release of growth hormone from the pituitary gland which plays an important role in energy metabolism (48, 49). Therefore, the current study shows that the level of ornithine is significantly increased after SADI-S.

Although this is the first study to explore the effects of SADI-S on metabolites of stool in T2DM rats, the study also had some limitations. First,we didn’t set other bariatric surgeries as control group of SADI-S surgery,so it is unclear whether other bariatric surgeries will also result in similar changes in fecal metabolomics as SADI-S surgery. Second,metabolites were only detected in feces of T2DM rats rather than serum metabolites.Third, the effects of SADI-S was not evaluated on metabolites in patients with T2DM. Therefore, there is need for further studies, especially clinical studies to be performed on the effects of SADI-S on serum metabolites in T2DM.

## Conclusion

The current study shows that SADI-S can improve the disease state of T2DM rats. Mechanistically, the improvement of T2DM caused by SADI-S may be associated with the changes of pathways such as tryptophan metabolism pathway, linoleic acid metabolism pathway and so on.

## Data availability statement

The original contributions presented in the study are included in the article/**Supplementary Material**. Further inquiries can be directed to the corresponding author.

## Ethics statement

The animal study was reviewed and approved by The Animal Experiment Ethics Committee of First hospital of Jilin University.

## Author contributions

LW: Conceptualization, Methodology, Software, Data Curation, Formal Analysis, Writing-Original Draft. ZW, YY, ZR, YJ, JW, and SL: Data Curation, Formal Analysis; TJ: Conceptualization, Funding Acquisition, Resources, Supervision, Writing - Review & Editing. All authors contributed to the article and approved the submitted version.

## Funding

The special health research talents project of Jilin province (2020SCZ04).

## Conflict of interest

The authors declare that the research was conducted in the absence of any commercial or financial relationships that could be construed as a potential conflict of interest.

## Publisher’s note

All claims expressed in this article are solely those of the authors and do not necessarily represent those of their affiliated organizations, or those of the publisher, the editors and the reviewers. Any product that may be evaluated in this article, or claim that may be made by its manufacturer, is not guaranteed or endorsed by the publisher.
