# Metabolomics analysis of stool in rats with type 2 diabetes mellitus after single-anastomosis duodenal–ileal bypass with sleeve gastrectomy

**DOI:** 10.3389/fendo.2023.1128339

**Published:** 2023-03-28

**Authors:** Lun Wang, Zeyu Wang, Yang Yu, Zhaoheng Ren, Yongheng Jia, Jinfa Wang, Shixing Li, Tao Jiang

**Affiliations:** Department of Bariatric and Metabolic Surgery, China-Japan Union Hospital of Jilin University, Changchun, China

**Keywords:** single-anastomosis duodenal–ileal bypass with sleeve gastrectomy, type 2 diabetes mellitus, metabolomics, bariatric surgery, SADI-S, T2DM

**Text Correction**

In the published article, there was an inappropriate description because of our limited metabolomics knowledge at the time of writing this paper. A correction has been made to **Abstract**, **Results**. This sentence previously stated:

“A total of 245 differential metabolites were identified between the two groups. Among them, 16 metabolites such as branched-chain amino acids (valine), aromatic amino acid (phenylalanine), bile acid (cholic acid, lithocholic acid, and β-muricholic acid), short-chain fatty acid (isobutyric acid), and phospholipid lysoPE(17:0), lysoPE(20:3) and lysoPS(16:0) were associated to the T2DM remission after SADI-S.”The corrected sentence appears below: “A total of 245 differential metabolites were identified between the two groups, among which 8 metabolites were detectable under both the positive ion model and negative ion model. Therefore, a total of 237 differential metabolites were identified in our study which were mainly involved in tryptophan metabolism; cysteine and methionine metabolism; phenylalanine metabolism; phenylalanine; tyrosine and tryptophan biosynthesis; arginine biosynthesis; alanine, aspartate and glutamate metabolism; Arginine and proline metabolism; glyoxylate and dicarboxylate metabolism; alpha-Linolenic acid metabolism; Linoleic acid metabolism; riboflavin metabolism; nicotinate and nicotinamide metabolism; pyrimidine metabolism; porphyrin and chlorophyll metabolism.”

**Text Correction**

In the published article, there was an inappropriate description because of our limited metabolomics knowledge at the time of writing this paper. Our study only found an effect of SADI-S surgery on the metabolites of stool in T2DM rats and did not further demonstrate that these different metabolites caused by SADI-S can improve or worsen T2DM. Therefore, we could not draw a direct causal association between the metabolites and the T2DM improvement caused by SADI-S. Consequently, our previous conclusion is not scientific.

A correction has been made to the **Abstract**, **Conclusion**. This sentence previously stated:

“SADI-S improves T2DM in rats by regulating phenylalanine biosynthesis, valine, phenylalanine, alanine, glutamate, proline, bile acid, and phospholipid metabolism pathways”

The corrected sentence appears below:

“SADI-S significantly improved the glucose metabolism in T2DM rats. In addition, SADI-S significantly changed the composition of metabolites in T2DM rats which were involved in tryptophan metabolism pathway, linoleic acid metabolism pathway and so on. This may be the mechanism by which SADI-S improved T2DM.”

**Text Correction**

In the published article, there was an inaccurate description. To supplement the description of results.

A correction has been made to **Results**, Identification of differential metabolites of Metabolomics analysis, Line 5-8 of Paragraph 1. This sentence previously stated:

“The data shown in the Supplemental Table 1 indicated that 245 differential metabolites were identified, among which 81 metabolites were obtained under the positive ion model and 164

metabolites were obtained under the negative ion model.”

The corrected sentence appears below:

“The data shown in the Supplemental Table 1 indicated that 245 differential metabolites were identified, among which 81 metabolites were obtained under the positive ion model and 164

metabolites were obtained under the negative ion model. Eight of these metabolites were detectable in both modes. Therefore, a total of 237 differential metabolites were identified between the two groups.”

**Text Correction**

In the published article, there was an inappropriate description because of our limited metabolomics knowledge at the time of writing this paper. We did not properly describe our results.

A correction has been made to the **Results**, Identification of differential metabolites of Metabolomics analysis, Line 12-17 of Paragraph. This sentence previously stated:

“Sixteen metabolites were associated to T2DM remission after SADI-S such as branched-chain amino acids (valine), aromatic amino acid (phenylalanine), bile acid (cholic acid, lithocholic acid and b-muricholic acid), short-chain fatty acid (isobutyric acid), phospholipid lysoPE(17:0), lysoPE (20:3), and lysoPS(16:0).”

The corrected sentence appears below:

“According to the previous literature, some metabolites that may be associated with the T2DM such as branched-chain amino acids (valine), aromatic amino acid (phenylalanine), bile acid (cholic acid, lithocholic acid and b-muricholic acid), short-chain fatty acid (isobutyric acid), phospholipid lysoPE(17:0), lysoPE (20:3), and lysoPS(16:0), were also observed to have a significant changes in our study.”

**Text Correction**

In the published article, there was an inappropriate description because of our limited metabolomics knowledge at the time of writing this paper. Our study only found an effect of SADI-S surgery on the metabolites of stool in T2DM rats and did not further demonstrate that these different metabolites caused by SADI-S can improve or worsen T2DM. Therefore, we could not draw a direct causal association between the metabolites and the T2DM improvement caused by SADI-S. Consequently, our previous conclusion is not scientific.

A correction has been made to the **Conclusion**. This sentence previously stated:

“The current study shows that SADI-S can improve the disease state of T2DM rats. Mechanistically, it regulates valine metabolism, phenylalanine metabolism, phenylalanine biosynthesis, alanine and glutamate metabolism, proline metabolism, bile acid metabolism as well as the phospholipid metabolism pathways”The corrected sentence appears below: “The current study shows that SADI-S can improve the disease state of T2DM rats. Mechanistically, the improvement of T2DM caused by SADI-S may be associated with the changes of pathways such as tryptophan metabolism pathway, linoleic acid metabolism pathway and so on.”

**Error in Figure/Table**

In the published article, there was an error in **Figures 1B** as published. We previously calculated the area under the curve (AUC) of IPGTT according to a formula described in a literature. However, at that time we did not discover that there were some differences between this literature and our research. Therefore, we used the wrong formula AUC = (G0 + G180)/2 + G15 + G30 + G60 + G120 to calculate the AUC of IPGTT. Now we calculated the AUC using the correct formula AUC=(G0+G15)×15/2+(G15+G30)×15/2+(G30+G60)×30/2+(G60+

**Figure 1B:**
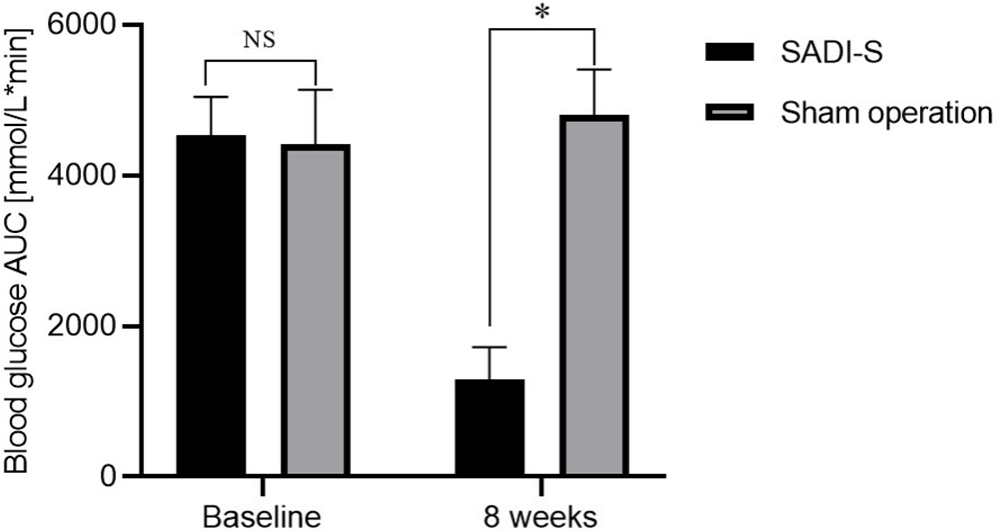
Changes in blood glucose AUC during IPGTT before and after surgery. NS indicates no significant difference between the SADI-S group and sham operation group. * represents a significant difference between the SADI-S group and sham operation group (P< 0.05).

G120)×60/2+(G120+G180)×60/2 and redrawn the **Figure 1B**. The corrected **Figures 1B** and its caption appear below. The authors apologize for these errors and state that they does not change the scientific conclusions of the article in any way. The original article has been updated.

