# Supplementary figures and images for: Metabolomics analysis of stool in rats with type 2 diabetes mellitus after single-anastomosis duodenal–ileal bypass with sleeve gastrectomy
Source: Front Endocrinol (Lausanne). 2022 Sep 20;13:1013959. doi: 10.3389/fendo.2022.1013959 (PMC9530139; doi:10.3389/fendo.2022.1013959)

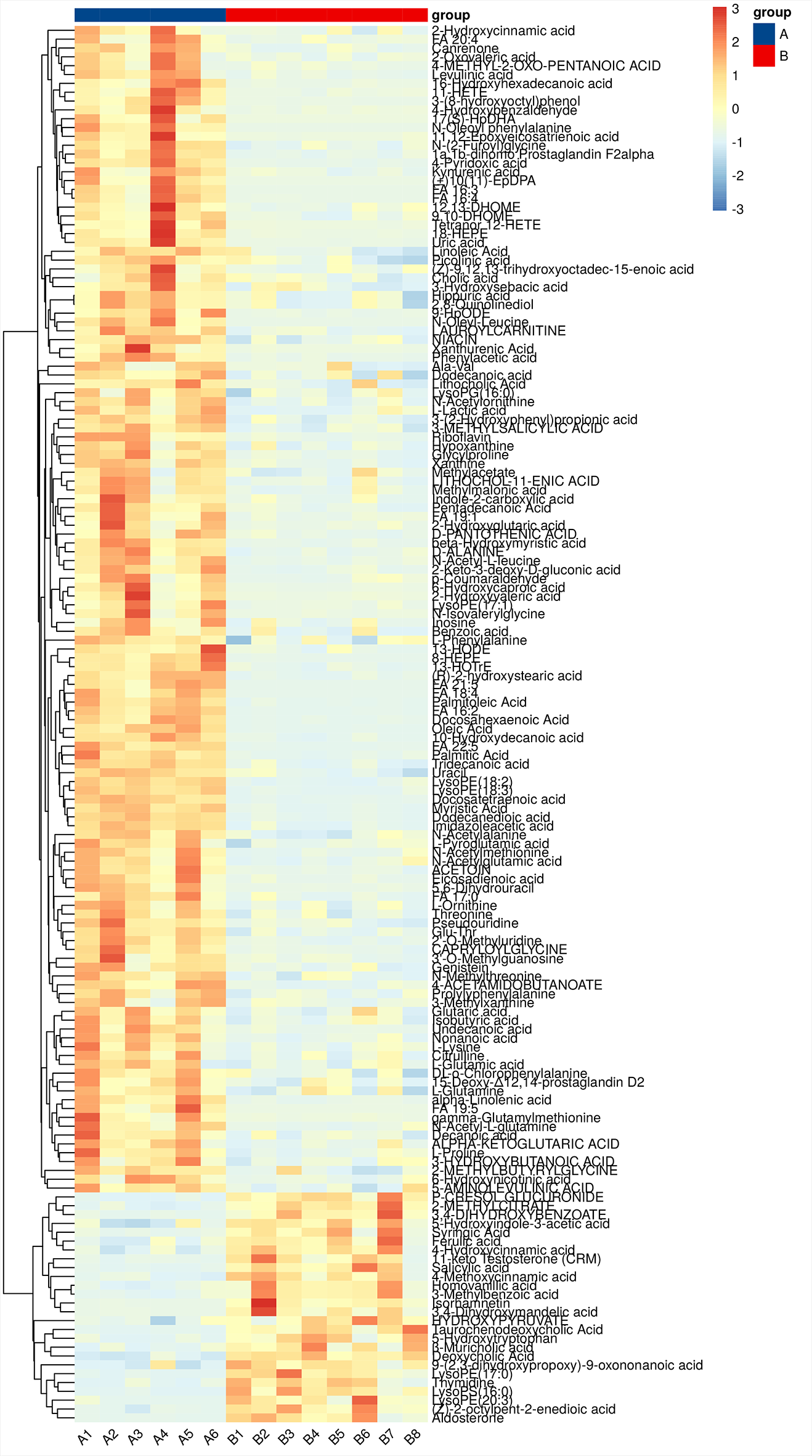

Supplement: Supplementary file 1 [file Image_1.tif]

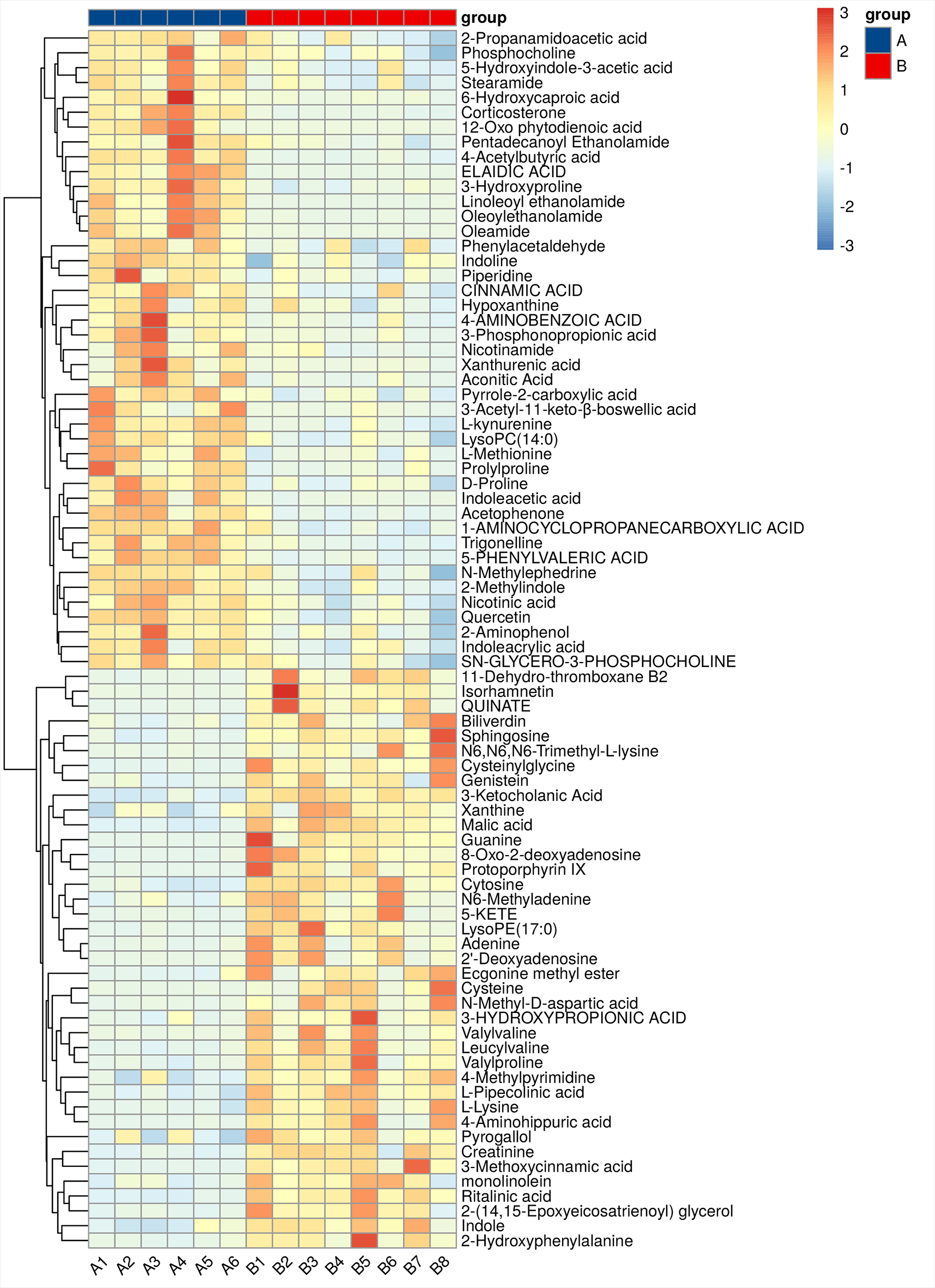

Supplement: Supplementary file 2 [file Image_2.tif]
