# Supplementary material for: Metabolomics analysis of stool in rats with type 2 diabetes mellitus after single-anastomosis duodenal–ileal bypass with sleeve gastrectomy
Source: Front Endocrinol (Lausanne). 2022 Sep 20;13:1013959. doi: 10.3389/fendo.2022.1013959 (PMC9530139; doi:10.3389/fendo.2022.1013959)
Supplement: Supplementary file 3 [file Table_1.doc]

Table 1 Differential metabolites between the SADI-S group and the sham operation group

| Mode | Metabolites | RT (min) | MS2.score | VIP | FC  (SADI-S/Sham operation) | Trend |
| --- | --- | --- | --- | --- | --- | --- |
| ESI- | FA 16:2 | 11.92 | 94.8 | 1.55 | 68.83 | ↑*** |
|  | alpha-Linolenic acid | 12.26 | 96.8 | 1.42 | 9.35 | ↑** |
|  | 13-Hode | 11.31 | 91.6 | 1.27 | 2.34 | ↑** |
|  | N-Oleoyl phenylalanine | 13.42 | 87 | 1.37 | 785.55 | ↑** |
|  | beta-Hydroxymyristic acid | 9.62 | 36.3 | 1.44 | 7.89 | ↑** |
|  | FA 16:3 | 11.31 | 95.8 | 1.33 | 3635.91 | ↑** |
|  | Pseudouridine | 0.85 | 89.9 | 1.39 | 2.94 | ↑** |
|  | FA 16:4 | 10.86 | 79.5 | 1.32 | 505.42 | ↑* |
|  | Lithocholic Acid | 10.71 | 92.7 | 1.16 | 3.22 | ↑** |
|  | Palmitoleic Acid | 12.63 | 99.9 | 1.55 | 10.60 | ↑*** |
|  | Pentadecanoic Acid | 12.79 | 93.5 | 1.36 | 5.06 | ↑** |
|  | Palmitic Acid | 13.42 | 95.8 | 1.51 | 9.05 | ↑*** |
|  | (R)-2-hydroxystearic acid | 11.49 | 91.2 | 1.52 | 14.54 | ↑*** |
|  | Glu-Thr | 0.81 | 60.7 | 1.47 | 2.30 | ↑*** |
|  | N-Oleyl-Leucine | 13.42 | 94.1 | 1.43 | 248.15 | ↑** |
|  | Cholic acid | 7.08 | 93.6 | 1.03 | 2.04 | ↑* |
|  | LysoPE(18:2) | 9.68 | 80.5 | 1.59 | 5.36 | ↑*** |
|  | LAUROYLCARNITINE | 11.27 | 16.5 | 1.41 | 3.50 | ↑** |
|  | Oleic Acid | 13.70 | 94.1 | 1.55 | 57.46 | ↑*** |
|  | LysoPE(17:0) | 10.75 | 92.8 | 1.26 | 0.10 | ↓** |
|  | 11,12-Epoxyeicosatrienoic acid | 10.55 | 78.7 | 1.13 | 17.09 | ↑* |
|  | 11-Hete | 10.47 | 89.6 | 1.20 | 8.10 | ↑* |
|  | 8-HEepe | 10.08 | 86.2 | 1.45 | 80.94 | ↑** |
|  | (Z)-2-octylpent-2-enedioic acid | 7.36 | 82.8 | 1.24 | 0.32 | ↓** |
|  | 18-Hepe | 9.89 | 69.5 | 1.20 | 17.35 | ↑* |
|  | (±)10(11)-EpDPA | 10.43 | 77.8 | 1.25 | 125.92 | ↑* |
|  | Linoleic Acid | 11.35 | 99.2 | 1.39 | 2.26 | ↑*** |
|  | Thymidine | 2.09 | 83.2 | 1.18 | 0.25 | ↓** |
|  | LysoPE(17:1) | 9.94 | 93.6 | 1.18 | 8.59 | ↑* |
|  | gamma-Glutamylmethionine | 2.17 | 97.6 | 1.12 | 32.37 | ↑* |
|  | β-Muricholic acid | 6.86 | 95.4 | 1.13 | 0.27 | ↓** |
|  | FA 22:4 | 13.72 | 92.5 | 1.04 | 530.75 | ↑*** |
|  | 3'-O-Methylguanosine | 1.91 | 93.3 | 1.23 | 5.97 | ↑* |
|  | FA 17:2 | 10.84 | 90.3 | 1.02 | 3.84 | ↑* |
|  | FA 22:5 | 12.87 | 91.5 | 1.56 | 53.19 | ↑*** |
|  | 2'-O-Methyluridine | 1.86 | 73 | 1.48 | 8.71 | ↑** |
|  | FA 21:5 | 12.51 | 96.3 | 1.46 | 2964.63 | ↑** |
|  | Tetranor 12-HETE | 8.80 | 64.5 | 1.12 | 16.05 | ↑* |
|  | Deoxycholic Acid | 8.66 | 93.4 | 1.50 | 0.15 | ↓*** |
|  | Lithochol-11-enic acid | 11.54 | 80.3 | 1.23 | 4.44 | ↑* |
|  | LysoPG(16:0) | 11.13 | 95.1 | 1.16 | 1.83 | ↑** |
|  | FA 18:4 | 11.71 | 90.4 | 1.47 | 1633.44 | ↑** |
|  | 16-Hydroxyhexadecanoic acid | 10.27 | 84.2 | 1.24 | 27.69 | ↑* |
|  | FA 19:1 | 14.38 | 93 | 1.22 | 8.23 | ↑* |
|  | Inosine | 1.66 | 56.8 | 1.04 | 2.75 | ↑* |
|  | FA 20:4 | 12.74 | 90.6 | 1.10 | 2.55 | ↑* |
|  | 12,13-Dhome | 8.81 | 94.2 | 1.06 | 3.12 | ↑* |
|  | Docosahexaenoic Acid | 12.56 | 89 | 1.52 | 21.80 | ↑*** |
|  | LysoPE(18:3) | 8.90 | 47.4 | 1.60 | 16.79 | ↑*** |
|  | Docosatetraenoic acid | 13.41 | 63.7 | 1.59 | 20.16 | ↑*** |
|  | 11-keto Testosterone (CRM) | 6.28 | 74.5 | 1.10 | 0.27 | ↓** |
|  | 3-(8-hydroxyoctyl)phenol | 10.49 | 73.6 | 1.33 | 10.10 | ↑** |
|  | (Z)-9,12,13-trihydroxyoctadec-15-enoic acid | 7.62 | 84.7 | 1.08 | 1.91 | ↑* |
|  | 17(S)-HpDHA | 9.80 | 82.1 | 1.07 | 153.81 | ↑*** |
|  | Prolylyphenylalanine | 3.90 | 11 | 1.35 | 2.32 | ↑** |
|  | 9-(2,3-dihydroxypropoxy)-9-oxononanoic acid | 4.59 | 31.1 | 1.15 | 0.41 | ↓** |
|  | 13-HOTrE | 9.82 | 82.7 | 1.46 | 5.22 | ↑** |
|  | 9-HpODE | 9.17 | 88.4 | 1.35 | 16.46 | ↑** |
|  | Taurochenodeoxycholic Acid | 7.32 | 93 | 1.25 | 0.01 | ↓** |
|  | Genistein | 5.36 | 92.8 | 1.36 | 3.70 | ↑** |
|  | 15-Deoxy-Δ12,14-prostaglandin D2 | 9.75 | 74.7 | 1.13 | 1.49 | ↑* |
|  | Xanthosine | 1.79 | 83.1 | 1.01 | 9.31 | ↑* |
|  | FA 19:5 | 11.80 | 37.3 | 1.21 | 665.21 | ↑* |
|  | Dodecanedioic acid | 7.13 | 92 | 1.61 | 22.09 | ↑*** |
|  | Eicosadienoic acid | 13.94 | 97.9 | 1.33 | 17.71 | ↑** |
|  | LysoPS(16:0) | 10.29 | 94.5 | 1.32 | 0.08 | ↓*** |
|  | 19,20-DiHDPA | 9.37 | 82.8 | 1.03 | 10.65 | ↑** |
|  | FA 17:0 | 14.01 | 97.5 | 1.36 | 3.99 | ↑** |
|  | Myristic Acid | 12.39 | 95.7 | 1.59 | 8.13 | ↑*** |
|  | 9,10-Dhome | 9.02 | 93.7 | 1.29 | 3.80 | ↑** |
|  | 1a,1b-dihomoProstaglandin F2alpha | 10.77 | 47.4 | 1.44 | 6.13 | ↑** |
|  | 5-Hydroxytryptophan | 1.31 | 54.9 | 1.17 | 0.13 | ↓** |
|  | P-cresol Glucuronide | 4.21 | 31.4 | 1.37 | 0.06 | ↓*** |
|  | Isorhamnetin | 6.07 | 82.6 | 1.02 | 0.04 | ↓* |
|  | LysoPE(20:3) | 9.98 | 87.2 | 1.06 | 0.08 | ↓** |
|  | Riboflavin | 3.95 | 94.7 | 1.32 | 5.43 | ↑** |
|  | 3-Hydroxysebacic acid | 4.50 | 52.2 | 1.11 | 2.02 | ↑* |
|  | D-Pantothenic Acid | 2.73 | 99 | 1.34 | 2.80 | ↑** |
|  | Tridecanoic acid | 11.68 | 89.6 | 1.57 | 5.07 | ↑*** |
|  | Syringic Acid | 4.14 | 96.6 | 1.07 | 0.31 | ↓** |
|  | 2-Methylcitrate | 1.35 | 68 | 1.31 | 0.13 | ↓*** |
|  | Capryloylglycine | 5.65 | 32.7 | 1.41 | 4.35 | ↑** |
|  | Xanthurenic Acid | 0.98 | 93.3 | 1.15 | 15.83 | ↑* |
|  | DL-o-Chlorophenylalanine | 3.27 | 89.9 | 1.23 | 1.33 | ↑** |
|  | 4-Methoxycinnamic acid | 4.10 | 72.9 | 1.22 | 0.29 | ↓** |
|  | Glycylproline | 1.22 | 16 | 1.42 | 2.97 | ↑** |
|  | Homovanillic acid | 3.54 | 79.6 | 1.18 | 0.19 | ↓** |
|  | N-Acetylmethionine | 3.47 | 67.5 | 1.38 | 7.08 | ↑** |
|  | N-Acetyl-L-glutamine | 1.21 | 15.1 | 1.29 | 4.19 | ↑** |
|  | Ferulic acid | 4.88 | 95 | 1.10 | 0.31 | ↓** |
|  | 10-Hydroxydecanoic acid | 6.03 | 57.2 | 1.56 | 5.58 | ↑*** |
|  | Ala-Val | 1.77 | 65.6 | 1.04 | 2.62 | ↑* |
|  | Kynurenic acid | 3.31 | 95.6 | 1.27 | 4.40 | ↑* |
|  | 3,4-Dihydroxymandelic acid | 3.30 | 90.3 | 1.01 | 0.06 | ↓* |
|  | N-Acetylglutamic acid | 0.85 | 89.8 | 1.28 | 3.82 | ↑** |
|  | Dodecanoic acid | 7.86 | 74.6 | 1.11 | 1.30 | ↑** |
|  | 5-Hydroxyindole-3-acetic acid | 4.37 | 93.3 | 1.18 | 0.33 | ↓** |
|  | Undecanoic acid | 10.51 | 91.5 | 1.33 | 9.83 | ↑** |
|  | 4-Pyridoxic acid | 1.22 | 83.8 | 1.46 | 14.97 | ↑** |
|  | N-Acetyl-L-leucine | 4.31 | 97.5 | 1.38 | 7.83 | ↑** |
|  | 2-Keto-3-deoxy-D-gluconic acid | 0.82 | 82.6 | 1.45 | 8.50 | ↑** |
|  | N-(2-Furoyl)glycine | 3.83 | 31.6 | 1.38 | 3.66 | ↑** |
|  | Citrulline | 0.78 | 67.8 | 1.14 | 2.38 | ↑* |
|  | 3-Methylbenzoic acid | 3.54 | 85 | 1.15 | 0.25 | ↓** |
|  | Decanoic acid | 9.79 | 72 | 1.21 | 2.13 | ↑* |
|  | Hippuric acid | 3.68 | 45 | 1.25 | 1.92 | ↑** |
|  | 3-Methylxanthine | 1.96 | 55.3 | 1.15 | 4.00 | ↑* |
|  | N-Acetylornithine | 0.81 | 67.4 | 1.41 | 3.26 | ↑** |
|  | 4-Hydroxycinnamic acid | 4.64 | 87.9 | 1.19 | 0.43 | ↓** |
|  | 3-(2-Hydroxyphenyl)propionic acid | 5.20 | 90.1 | 1.45 | 4.03 | ↑*** |
|  | 4-Acetamidobutanoate | 0.84 | 25.2 | 1.40 | 2.40 | ↑** |
|  | L-Phenylalanine | 2.24 | 92.3 | 1.22 | 1.83 | ↑** |
|  | Indole-2-carboxylic acid | 4.23 | 97.6 | 1.13 | 5.65 | ↑* |
|  | 2,8-Quinolinediol | 3.70 | 97.7 | 1.25 | 1.94 | ↑** |
|  | Alpha-Ketoglutaric Acid | 0.85 | 76.1 | 1.19 | 2.51 | ↑* |
|  | 2-Methylbutyrylglycine | 4.28 | 49.1 | 1.25 | 2.22 | ↑*** |
|  | N-Isovalerylglycine | 3.77 | 80.6 | 1.20 | 8.25 | ↑* |
|  | Uric acid | 0.85 | 90.4 | 1.31 | 134.14 | ↑* |
|  | 3,4-Dihydroxybenzoate | 3.05 | 98.4 | 1.14 | 0.07 | ↓** |
|  | Aminoadipic Acid | 1.68 | 46.8 | 1.02 | 1.83 | ↑** |
|  | Nonanoic acid | 9.03 | 62.7 | 1.36 | 2.56 | ↑** |
|  | 2-Hydroxyglutaric acid | 0.85 | 91.9 | 1.12 | 4.18 | ↑* |
|  | 3-Methylsalicylic Acid | 4.53 | 87.2 | 1.50 | 3.35 | ↑*** |
|  | L-Glutamine | 0.77 | 24.9 | 1.02 | 1.74 | ↑* |
|  | 6-Hydroxynicotinic acid | 1.22 | 92.7 | 1.21 | 2.72 | ↑* |
|  | L-Lysine | 0.73 | 92.5 | 1.18 | 3.10 | ↑* |
|  | Xanthine | 1.21 | 94.9 | 1.42 | 4.15 | ↑** |
|  | p-Coumaraldehyde | 4.80 | 80.1 | 1.13 | 6.24 | ↑* |
|  | 2-Methylglutaric acid | 1.76 | 78.4 | 1.01 | 27.92 | ↑*** |
|  | L-Glutamic acid | 0.78 | 88.7 | 1.16 | 2.31 | ↑* |
|  | N-Methylthreonine | 0.77 | 56.2 | 1.38 | 3.20 | ↑*** |
|  | N-Acetylalanine | 0.79 | 33.2 | 1.43 | 1.55 | ↑*** |
|  | Salicylic acid | 4.18 | 98.5 | 1.20 | 0.24 | ↓** |
|  | L-Pyroglutamic acid | 0.84 | 90.9 | 1.29 | 2.39 | ↑** |
|  | Phenylacetic acid | 5.60 | 81.6 | 1.40 | 9.53 | ↑** |
|  | 5-Aminolevulinic Acid | 1.38 | 31.2 | 1.22 | 2.36 | ↑** |
|  | Hypoxanthine | 0.85 | 99.1 | 1.27 | 2.82 | ↑** |
|  | Picolinic acid | 1.21 | 84 | 1.25 | 1.74 | ↑** |
|  | Glutaric acid | 1.93 | 96.7 | 1.07 | 2.40 | ↑* |
|  | L-Ornithine | 0.78 | 68.9 | 1.26 | 2.65 | ↑** |
|  | 4-Methyl-2-OXO-Pentanoic Acid | 3.51 | 88.6 | 1.13 | 5.58 | ↑* |
|  | 6-Hydroxycaproic acid | 4.35 | 91.3 | 1.26 | 32.93 | ↑* |
|  | 5,6-Dihydrouracil | 0.87 | 64.8 | 1.35 | 30.27 | ↑** |
|  | L-Proline | 0.82 | 58.3 | 1.30 | 2.61 | ↑** |
|  | Benzoic acid | 4.21 | 89 | 1.17 | 2.40 | ↑** |
|  | Imidazoleacetic acid | 1.51 | 94.9 | 1.59 | 7.90 | ↑*** |
|  | 4-Hydroxybenzaldehyde | 4.42 | 97.1 | 1.13 | 6.88 | ↑* |
|  | NIACIN | 0.91 | 85.6 | 1.41 | 1.87 | ↑*** |
|  | Threonine | 0.78 | 69.5 | 1.34 | 2.56 | ↑** |
|  | Canrenone | 13.41 | 49.9 | 1.34 | 4.33 | ↑** |
|  | 2-Oxovaleric acid | 1.56 | 79.8 | 1.12 | 3.65 | ↑* |
|  | Levulinic acid | 1.95 | 86.3 | 1.33 | 8.53 | ↑** |
|  | 2-Hydroxyvaleric acid | 3.22 | 87.6 | 1.10 | 12.57 | ↑* |
|  | Aldosterone | 7.50 | 80.2 | 1.16 | 0.14 | ↓** |
|  | Hydroxypyruvate | 1.21 | 67.6 | 1.09 | 0.51 | ↓** |
|  | Methylmalonic acid | 0.95 | 94.3 | 1.21 | 5.40 | ↑* |
|  | Uracil | 1.21 | 93.6 | 1.52 | 2.56 | ↑*** |
|  | 3-Hydroxybutanoic Acid | 1.44 | 89.2 | 1.08 | 2.50 | ↑* |
|  | Acetoin | 3.87 | 92.2 | 1.40 | 6.51 | ↑** |
|  | L-Lactic acid | 1.21 | 89.5 | 1.31 | 5.02 | ↑** |
|  | Isobutyric acid | 1.93 | 91.4 | 1.21 | 3.04 | ↑* |
|  | D-Alanine | 0.77 | 91.6 | 1.43 | 3.82 | ↑** |
|  | 2-Hydroxycinnamic acid | 4.21 | 11.4 | 1.05 | 1.86 | ↑* |
|  | Methylacetate | 0.95 | 83 | 1.25 | 6.03 | ↑* |
| ESI+ | Nicotinic acid | 1.22 | 98.2 | 1.35 | 1.88 | ↑** |
|  | Phenylacetaldehyde | 1.24 | 44.2 | 1.02 | 1.49 | ↑* |
|  | 2-Propanamidoacetic acid | 1.22 | 26.3 | 1.16 | 1.47 | ↑** |
|  | Indoline | 2.25 | 91.7 | 1.28 | 1.71 | ↑** |
|  | Nicotinamide | 0.84 | 49.7 | 1.10 | 1.83 | ↑* |
|  | L-Methionine | 0.85 | 19.9 | 1.33 | 2.12 | ↑** |
|  | Adenine | 1.40 | 98.9 | 1.10 | 0.08 | ↓* |
|  | Cysteine | 4.40 | 36.3 | 1.03 | 0.02 | ↓* |
|  | 3-Hydroxyproline | 0.82 | 50.8 | 1.37 | 1.95 | ↑** |
|  | L-Lysine | 0.75 | 94.6 | 1.30 | 0.35 | ↓** |
|  | L-Pipecolinic acid | 0.75 | 96.4 | 1.43 | 0.35 | ↓*** |
|  | 6-Hydroxycaproic acid | 4.20 | 19.9 | 1.09 | 3.56 | ↑* |
|  | Pyrrole-2-carboxylic acid | 0.84 | 52.9 | 1.35 | 1.35 | ↑** |
|  | Indole | 4.38 | 99 | 1.28 | 0.34 | ↓** |
|  | Indoleacetic acid | 2.60 | 63 | 1.34 | 8.92 | ↑* |
|  | 2-Methylindole | 0.85 | 30.4 | 1.50 | 1.72 | ↑*** |
|  | Xanthine | 0.85 | 73.2 | 1.11 | 0.57 | ↓** |
|  | Guanine | 1.50 | 98.7 | 1.17 | 0.03 | ↓** |
|  | Creatinine | 0.81 | 95.7 | 1.36 | 0.20 | ↓*** |
|  | 4-Aminohippuric acid | 1.43 | 22.9 | 1.14 | 0.05 | ↓** |
|  | Acetophenone | 1.39 | 83.5 | 1.48 | 6.43 | ↑** |
|  | Ecgonine methyl ester | 1.61 | 69.2 | 1.10 | 0.26 | ↓** |
|  | D-Proline | 0.84 | 98.3 | 1.43 | 1.58 | ↑*** |
|  | 2-Hydroxyphenylalanine | 1.90 | 85.2 | 1.09 | 0.27 | ↓** |
|  | Valylvaline | 1.93 | 50.2 | 1.00 | 0.14 | ↓* |
|  | N-Methyl-D-aspartic acid | 6.28 | 44.3 | 1.10 | 0.05 | ↓* |
|  | 4-Acetylbutyric acid | 11.46 | 38 | 1.51 | 3.74 | ↑** |
|  | 4-Aminobenzoic Acid | 2.28 | 30.8 | 1.19 | 3.07 | ↑* |
|  | Xanthurenic acid | 0.99 | 91.2 | 1.15 | 3.99 | ↑* |
|  | Indoleacrylic acid | 3.92 | 94 | 1.19 | 1.51 | ↑* |
|  | Trigonelline | 1.22 | 59.4 | 1.47 | 3.28 | ↑*** |
|  | 5-Phenylvaleric Acid | 4.79 | 63.1 | 1.52 | 6.48 | ↑*** |
|  | Cysteinylglycine | 9.84 | 92.1 | 1.27 | 0.08 | ↓** |
|  | 3-Methoxycinnamic acid | 4.19 | 95.4 | 1.26 | 0.09 | ↓** |
|  | Leucylvaline | 2.97 | 33.8 | 1.21 | 0.15 | ↓** |
|  | Valylproline | 3.67 | 41.4 | 1.19 | 0.21 | ↓** |
|  | N6-Methyladenine | 2.54 | 66.6 | 1.01 | 0.27 | ↓* |
|  | 5-Hydroxyindole-3-acetic acid | 4.28 | 98.8 | 1.19 | 3.25 | ↑** |
|  | 6-Aminopenicillanic acid | 4.88 | 27 | 1.07 | 589.76 | | ↑*** | | --- | |
|  | Cytosine | 0.81 | 97.3 | 1.42 | 0.39 | ↓*** |
|  | Prolylproline | 0.85 | 81.3 | 1.29 | 1.83 | ↑* |
|  | N-Methylephedrine | 9.16 | 64.7 | 1.12 | 1.29 | ↑** |
|  | L-kynurenine | 0.85 | 17.7 | 1.48 | 1.77 | ↑*** |
|  | 3-Phosphonopropionic acid | 10.03 | 19.5 | 1.14 | 4.04 | ↑* |
|  | 8-Oxo-2-deoxyadenosine | 1.51 | 69.5 | 1.22 | 0.11 | ↓** |
|  | Cinnamic Acid | 4.24 | 39.7 | 1.14 | 2.32 | ↑** |
|  | Ritalinic acid | 5.17 | 56.8 | 1.44 | 0.08 | ↓*** |
|  | LysoPE(17:0) | 10.74 | 37.9 | 1.20 | 0.09 | ↓** |
|  | 2'-Deoxyadenosine | 1.40 | 99.8 | 1.06 | 0.03 | ↓* |
|  | Phosphocholine | 8.42 | 81.9 | 1.02 | 1.29 | ↑* |
|  | LysoPC(14:0) | 8.98 | 70.6 | 1.41 | 1.53 | ↑*** |
|  | Genistein | 5.26 | 96.4 | 1.14 | 0.27 | ↓** |
|  | 2-Aminophenol | 7.64 | 71.2 | 1.19 | 1.36 | ↑** |
|  | Pentadecanoyl Ethanolamide | 9.01 | 65.4 | 1.13 | 2.14 | ↑* |
|  | Protoporphyrin IX | 12.90 | 76.2 | 1.19 | 0.05 | ↓** |
|  | Sphingosine | 8.71 | 64 | 1.27 | 0.23 | ↓** |
|  | Stearamide | 9.33 | 77.5 | 1.20 | 2.39 | ↑** |
|  | SN-Glycero-3-Phosphocholine | 7.88 | 93.4 | 1.08 | 1.25 | ↑** |
|  | Isorhamnetin | 6.07 | 93.1 | 1.01 | 0.04 | ↓* |
|  | Oleoylethanolamide | 12.39 | 96 | 1.34 | 21.70 | ↑** |
|  | Monolinolein | 12.15 | 62 | 1.15 | 0.36 | ↓** |
|  | Linoleoyl ethanolamide | 11.55 | 96.8 | 1.38 | 18.12 | ↑** |
|  | Oleamide | 12.07 | 95.8 | 1.28 | 18.95 | ↑* |
|  | Biliverdin | 8.25 | 89.5 | 1.08 | 0.25 | ↓* |
|  | Elaidic Acid | 11.50 | 68.6 | 1.39 | 117.04 | ↑** |
|  | 3-Ketocholanic Acid | 8.66 | 79.8 | 1.60 | 0.12 | ↓*** |
|  | 1-Aminocyclopropanecarboxylic Acid | 1.23 | 72.7 | 1.38 | 2.07 | ↑*** |
|  | 4-Methylpyrimidine | 0.71 | 89.6 | 1.24 | 0.36 | ↓** |
|  | Piperidine | 0.89 | 74.4 | 1.14 | 1.67 | ↑* |
|  | 3-Hydroxypropionic Acid | 0.80 | 21.7 | 1.00 | 0.33 | ↓* |
|  | 3-Acetyl-11-keto-β-boswellic acid | 7.35 | 98.6 | 1.13 | 5.85 | ↑* |
|  | Aconitic Acid | 1.75 | 16.6 | 1.28 | 5.39 | ↑* |
|  | 2-(14,15-Epoxyeicosatrienoyl) glycerol | 11.11 | 79.3 | 1.19 | 0.16 | ↓** |
|  | 11-Dehydro-thromboxane B2 | 6.65 | 34 | 1.12 | 0.23 | ↓** |
|  | Malic acid | 0.85 | 18.3 | 1.46 | 0.21 | ↓*** |
|  | 5-KETE | 7.46 | 40.4 | 1.13 | 0.19 | ↓** |
|  | Corticosterone | 10.06 | 95.4 | 1.47 | 9.24 | ↑** |
|  | 12-Oxo phytodienoic acid | 10.88 | 57.9 | 1.24 | 115.64 | ↑* |
|  | Quinate | 9.76 | 16.7 | 1.13 | 0.01 | ↓** |
|  | Quercetin | 6.11 | 89.2 | 1.41 | 1.44 | ↑*** |
|  | N6,N6,N6-Trimethyl-L-lysine | 8.22 | 58.1 | 1.06 | 0.20 | ↓* |

FC=fold change;VIP=Variable Importance for the Projection;RT=retention time;FA=fatty acid;↑indicates increase; ↓indicates decrease;*P < 0.05;**P < 0.01;***P < 0.001.
